# HIV and ART status at baseline are associated longitudinally with increased pulse wave velocity: findings from the Ndlovu Cohort Study

**DOI:** 10.1097/QAD.0000000000004428

**Published:** 2026-02-03

**Authors:** Patane S. Shilabye, Karine Scheuermaier, Chijioke N. Umunnakwe, Roos E. Barth, Walter Devillé, Roel A. Coutinho, Diederick E. Grobbee, Willem D.F. Venter, Hugo Tempelman, Alinda G. Vos-Seda, Kerstin Klipstein-Grobusch

**Affiliations:** a Julius Global Health, Department of Global Public Health and Bioethics, Julius Center for Health Sciences and Primary Care, University Medical Center Utrecht, Utrecht University, Utrecht, The Netherlands; b Wits Sleep Lab, Brain Function Research Group, Department of Physiology, School of Biomedical Sciences, Faculty of Health Sciences, University of the Witwatersrand, Johannesburg; c Ndlovu Research Centre, Ndlovu Care Group, Elandsdoorn, Dennilton, South Africa; d Department of Infectious Diseases, University Medical Center Utrecht, Utrecht University, Utrecht; e PharmAccess Foundation, Amsterdam, The Netherlands; f Ezintsha, Faculty of Health Sciences, University of the Witwatersrand, Johannesburg; g Department of Public Health Medicine, School of Health Systems and Public Health, Faculty of Health Sciences, University of Pretoria, Pretoria; h School of Public Health, Faculty of Health Sciences, University of the Witwatersrand, Johannesburg, South Africa.

**Keywords:** antiretroviral therapy, cardiovascular disease risk, HIV, pulse wave velocity, South Africa

## Abstract

**Objective(s):**

People with HIV (PWH) have an increased risk of cardiovascular disease (CVD), but longitudinal data from middle-income settings remain limited. This study examined the association between HIV, antiretroviral therapy (ART), and pulse wave velocity (PWV), a marker of arterial stiffness and CVD risk.

**Design:**

A longitudinal analysis from the Ndlovu Cohort Study, South Africa.

**Methods:**

The study included 705 participants (325 PWH, 81% on ART at baseline, 19% initiating ART at baseline, and 380 HIV-negative people. Demographic data, HIV/ART status, and covariates were collected at baseline, while PWV was measured at 12 and 36  months. Mixed-effects models were used to analyze PWV changes over time, adjusting for age, sex, and systolic blood pressure (SBP). Results were reported as beta coefficients (*β*) with 95% confidence intervals (CIs).

**Results:**

At baseline, PWH were older and predominantly female (67%) compared to HIV-negative people. At 12  months, median PWV was higher in PWH (7.3  m/s) than in HIV-negative people (7.0  m/s, *P* = 0.001). Over 36  months, PWV increased by 0.30  m/s in PWH and 0.20 m/s in HIV-negative people (*P* = 0.002). ART-naïve individuals had the largest PWV increase after starting ART (6.8  m/s at 12  months to 7.4  m/s at 36  months, *P* = 0.001). HIV (*β* = 0.65, 95% CI: 0.24–1.06, *P* = 0.002) and time (*β* = 0.31 m/s per year, *P* < 0.001) were significantly associated with higher PWV.

**Conclusions:**

PWV increased over time, particularly in PWH, with ART initiation linked to rapid increases. These findings highlight the need for early CVD risk monitoring, especially post-ART initiation, in resource-limited settings.

## Introduction

Availability of antiretroviral treatment (ART) has significantly contributed to reduced disease progression and a nearly normal life expectancy of people with HIV (PWH) [1,2]. The increase in life expectancy, however, has led to the emergence of chronic age-related diseases particularly those affecting the cardiovascular system [2,3]. Previous research has demonstrated a two-fold increase in the risk of cardiovascular disease (CVD) among PWH compared to the general population [2,4,5]. Despite the growing body of evidence in this area, the precise mechanisms underlying CVD in PWH remain unclear. It is believed to be a complex multifactorial pathway involving HIV-induced inflammation as the key factor [4,6,7].

HIV-induced inflammation is considered to contribute to atherosclerosis and arterial stiffness by driving vascular smooth muscles to shift from a contractile to a pro-inflammatory and synthetic phenotype. This phenotypic modulation leads to increased secretion of inflammatory mediators, extracellular matrix remodeling, and reduced vascular compliances, all of which promote arterial stiffness and atherosclerotic plaque formation [8,9]. Predicting CVD risk is crucial for mitigating morbidity and mortality from CVD in PWH [10]. Studies conducted in high-income countries have highlighted the significant potential of pulse wave velocity (PWV) in providing information about early atherosclerosis disease burden. PWV, a noninvasive surrogate marker of CVD used to measure arterial stiffness has been shown to be highly predictive for CVD events in Caucasian populations in high-income countries [11,12] and therefore, has been considered a gold standard measure of arterial stiffness [13,14]. Furthermore, PWV can easily be obtained by measuring the carotid pulse pressure (cPP) and femoral pulse pressure (fPP), and the time delay between these pressures [10].

In South Africa, a few studies have cross-sectionally examined PWV among PWH and HIV-negative people in different provinces including North West, Western Cape, and Eastern Cape [15,16]. While these studies provide valuable insights, they remain limited in number and scope. Strategies to mitigate the relatively high burden of CVD risk in PWH have not been explored extensively [15–17]. Our study conducted in a rural population in Limpopo province, South Africa, provides a critical advancement by using longitudinal data to assess trends in PWV across a 36-month follow-up period. This approach enables a deeper understanding of disease progression and influence of ART on vascular health. By examining the temporal relationship between HIV status, ART, and arterial stiffness in a rural, resource-limited population, our study aimed to: assess longitudinal changes in PWV over a 36-month period in PWH and HIV-negative people; and examine the associations between PWV, HIV status, and ART status.

## Methods

This study is based on the Ndlovu cohort study (NCS), a prospective study from the Moutse area, Elandsdoorn, Sekhukhune, Limpopo Province, South Africa to investigate the relationship of HIV and ART and CVD risk in rural areas [18,19]. The study design is reported elsewhere in more detail [19]. In summary, recruitment took place between 2014 and 2016. Participants were residents living within approximately a 30 km radius of the Ndlovu Research Centre. Participants were recruited through community campaigns, at local events and shopping centers, as well as at the Ndlovu Medical Center, a large rural HIV treatment facility [18].

**Fig. 1:**
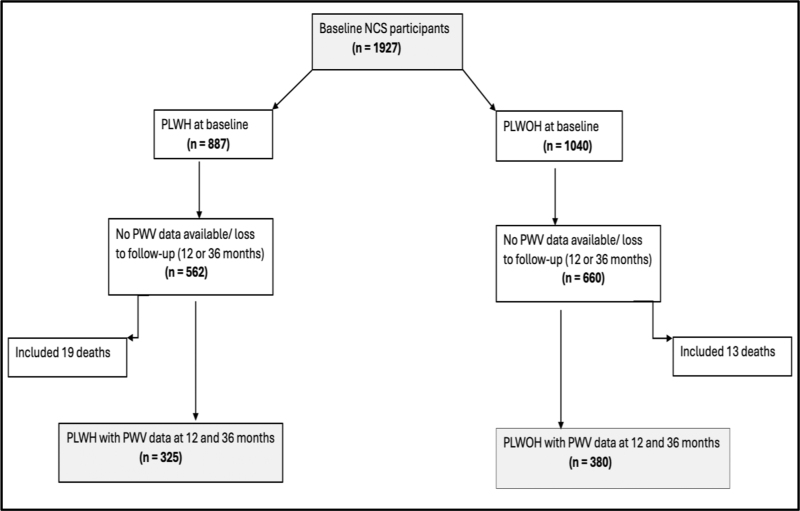
Study flow chart.

All participants underwent HIV testing at baseline and subsequent annual follow-up visits, with those who tested positive enrolled in the PWH group and being referred to the Ndlovu Medical Center of any other local HIV treatment facility, to initiate ART. Participants who tested positive were enrolled in the PWH group and referred to the Ndlovu Medical Center or another local HIV treatment facility to initiate ART. In addition, PWV was measured at the 12 and 36 month follow up visits. At baseline, 1927 participants were enrolled, of which 887 were PWH and 1040 were HIV-negative people. About 55% were women. Of the 690 PWH on ART, 613 (89%) were on 1^st^ line ART and 77 (11%) were on second line ART. At 12 months, there were 1445 participants who came for their follow-up (out of the 1927 at the beginning) and we were able to measure PWV in 1010 (507 PWH). At 36  months, 1190 came for their follow up and we had PWV measured in 1156 (482 PWH). In the current analysis, we included 325 PWH and 380 HIV-negative people aged 18  years and older, who had PWV data available at 12 and 36  months (Fig. 1).

Participants were excluded if they had missing pulse wave velocity (PWV) data at either the 12- or 36-month visit and/or were lost to follow-up. For people with HIV (PWH; *n* = 562) and HIV-negative (*n* = 660), exclusions were due to missed visits, incomplete measurements, withdrawal, or death. By the end of 36  months, 32 deaths had occurred (19 PWH and 13 HIV-negative).

Ethical clearance for the study was obtained from the ethics committees of the University of Pretoria (Ref. No.: 227/2014) and the University of the Witwatersrand (Ref. No.: M230850). Before enrolment, all participants provided written consent for study inclusion and data collection.

### Demographics and physical measurements

General characteristics such as age, sex, tobacco use, alcohol consumption, education, employment, socioeconomic status, and HIV-specific data including time on ART, duration of diagnosed HIV infection, stigma, and ART medication were obtained using a validated health questionnaire. ART was self-reported and complemented with ART information from TierNet [18], the electronic HIV registry. ART regimen was further classified at each time point as either first-line or second-line therapy for those on treatment. For participants who were ART-naïve at baseline, regimen data were available starting at 12  months. Standardized procedures were used for anthropometric (height, weight, hip, and waist circumference) and other physical measurements such as blood pressure and pulse rate at baseline and follow-up visits by well trained nurses.

### Biomedical analysis

Participants were instructed to fast overnight, after which blood samples were collected in the morning both at baseline and follow-up visits at the study site. The blood samples were used to measure glucose and lipids at baseline. Viral load and CD4^+^ were measured by trained laboratory technicians at baseline, and follow-up.

### Cardiovascular measurements

Carotid-femoral PWV, a measure of arterial stiffness expressed as the speed of pressure wave travelling through the arterial system in meters per second, was performed with the participants in a supine position, using a Sphygmocor (SphygmoCor XCEL, AtCor Medical PTY Ltd, Sydney, Australia) [20] at 12 and 36  months by well trained nurses. A higher PWV indicates greater arterial stiffness, which is a strong predictor of CVD risk, with a 1 m/s increase associated with a 1.14-fold increase in cardiovascular events. Detailed information on the methods associated with those PWV measurements has been previously published by Vos and colleagues [19]. Participants who came for the 12-month visit before 2016 did not have their PWV data collected because PWV measurements were only performed from 2016 onwards.

### Statistical analysis

Statistical analysis was conducted on the data of NCS participants who had PWV measurements at two time points (12 and 36  months) using R (version 4.4.0), via the RStudio integrated development environment [21,22]. Baseline data included independent variables such as HIV and ART status, age, sex, education, employment, monthly income per person, BMI, pulse rate per minute, triglycerides, total cholesterol, HDL, LDL, glucose, tobacco use, alcohol consumption, viral load and CD4^+^ cell count (Supplemantary Fig. 1, Supplemental Digital Content).

Descriptive analysis was conducted on baseline data for all participants with PWV data available. Continuous variables with normal distribution were reported as mean and standard deviation, skewed data as median with interquartile range. Categorical data were reported with numbers and percentages. The 325 PWH were categorized either as ART naïve at baseline or on ART at baseline. Pulse wave velocity was used as a continuous variable. Bivariable analysis was carried out to assess the association between various baseline variables including HIV and ART status, with PWV. The Kruskal–Wallis test was used to test for difference between HIV subgroups at each time point, followed by pairwise comparison using Wilcoxon rank sum test.

In addition, baseline characteristics were compared by HIV status (positive vs. negative) using Chi square or Fisher's exact tests for categorical variables, and *t*-tests or Wilcoxon rank sum tests for continuous variables, as appropriate. Standardized mean differences were also reported to aid interpretation. These results are provided in Table S1, Supplemental Digital Content.

To evaluate longitudinal changes in PWV, linear mixed models were applied. Confounding variables such as age, sex, education, systolic blood pressure (SBP) and triglycerides were selected based on their strength of association with PWV observed in the bivariate analyses. Multicollinearity was assessed among the variables using Variance Inflation Factor values and correlation matrix. Variables with multicollinearity were excluded from the linear mixed models. PWV trends over time were assessed using two classification methods: by HIV status at baseline (HIV-positive, HIV-negative) and by antiretroviral therapy (ART) status (HIV-negative, HIV-positive on ART, and HIV-positive and ART naïve at baseline). The ART regimen classification (first-line vs. second-line) was not modelled as an independent predictor due to small subgroup sizes and limited regimen detail. The final mixed models for both classification approaches were estimated using restricted maximum likelihood. Fixed effects included in the models were HIV groups, visit time (with 12  months as the reference category), age group (18–29 years as the reference), sex (female as the reference), education level (<high school as the reference), employment status (unemployed, as the reference), and as continuous variables SBP and total cholesterol. The results are expressed as parameter estimates with 95% confidence intervals (CIs). A *P*-value of <0.05 was considered to indicate statistical significance.

## Results

At baseline, 19% (*n* = 62) of the 325 PWH were ART-naïve. The mean age of participants (ART-naïve at baseline, PWH on ART and HIV-negative people) was 37.5 ± 10.6, 41.9 ± 9.9, and 36.5 ± 13.3  years, respectively, with significant difference between the groups (*P* < 0.001). The majority of participants were female (67%) in the PWH group, whereas in the HIV-negative people, males were the majority (63%) (Table 1). Mean SBP was higher in HIV-negative people (122.0 ± 23.9  mmHg) compared to ART naïve at baseline and PWH on ART (114.3 ± 20.3  mmHg, and 114.1 ± 18.6  mmHg) (Table 2). The PWV analytic sample was generally comparable to the full NCS cohort in education, employment, and cardiovascular risk factors. Minor differences included a slightly higher proportion of females in the HIV-positive sample and more males in the HIV-negative sample. Income and socioeconomic characteristics were similar, supporting the representativeness and generalizability of our findings despite small demographic variations. A more detailed breakdown of baseline characteristics, including lipid levels, smoking, and alcohol consumption by HIV status, is provided in Table S1, Supplemental Digital Content.

**Table 1:** Demographics and socioeconomic characteristics of the 705 NCS (Ndlovu Cohort Study) participants at baseline.

	PWH (*n* = 325)		
	ART naïve at baseline (*n* = 62)	On ART (*n* = 263)	HIV-negative (*n* = 380)
Demographics			
Age, years			
Mean (SD)	37.5 (10.6)	41.9 (9.9)	36.5 (13.3)
18–29 (%)	15 (24)	26 (9.5)	153 (40)
30-49	37 (60)	175 (67)	143 (38)
>49	10 (16)	62 (23.5)	84 (22)
Sex			
Female	45 (73)	172 (65)	142 (37)
Male	17 (27)	91 (35)	238 (63)
Socioeconomic characteristics			
Education			
Less than high school	16 (25)	62 (24)	69 (18)
High school/matric (HS)	40 (65)	190 (72)	275 (72)
Higher education (>HS)	6 (10)	11 (4)	36 (9)
Employment			
Unemployed	13 (21)	73 (28)	61 (16)
Employed	45 (73)	179 (68)	258 (68)
Other (student, retired, or volunteer)	4 (6)	11 (4)	61 (16)
Monthly income per person			
Median (IQR)	222.50 (0.00_925.00)	377.20 (0.00_1125.00)	341.70 (0.00_1312.50)
<648 ZAR	43 (69.4)	161 (62)	236 (62.1)
648–992 ZAR	4 (6.5)	25 (9.5)	40 (10.5)
>992 ZAR	15 (24.1)	75 (28.8)	104 (27.4)

ART, antiretroviral therapy; HS, high school; IQR, interquartile range; PWH, people with HIV; SD, standard deviation; ZAR, South African Rand (approximately 1 USD ≈ 18.4 ZAR at the time of study).

Note: Median monthly income per person in ZAR and approximate equivalent in USD:.

PWH (ART-naïve at baseline): 222.50 ZAR (≈ 12 USD), PWH on ART: 377.20 ZAR (≈ 21 USD), HIV-negative: 341.70 ZAR (≈ 19 USD).

Statistical measures: Mean (SD) reported for normally distributed continuous variables. Median (IQR) reported for skewed continuous variables. Percentages are shown for categorical variables.

**Table 2:** Clinical and lifestyle characteristics of 705 NSC (Ndlovu Cohort Study) participants at baseline.

	ART naïve at baseline (*n* = 62)	On ART (*n* = 263)	HIV-negative (*n* = 380)
Health status			
BMI (mean, SD): kg/m^2^	23.71 (5.94)	24.08 (5.87)	23.71 (5.23)
Systolic BP (mmHg)	114.20 (20.26)	114.41 (18.85)	122.05 (23.92)
Diastolic BP (mmHg)	74.58 (12.04)	73.38 (12.26)	75.19 (14.66)
Pulse rate per min (beats/min)	77.00 (68.00_87.75)	77.00 (67.00_80.00)	72.00 (64.00_78.00)
Triglycerides **(**mg/L)	0.90 (0.63_1.10)	1.00 (0.70_1.50)	0.90 (0.70_1.30)
Total cholesterol (mmol/L)	3.95 (3.40_4.40)	4.10 (3.70_4.80)	4.10 (3.50_4.70)
HDL (mmol/L)	1.31 (1.01_1.63)	1.46 (1.21_1.70)	1.38 (1.12_1.58)
LDL (mmol/L)	2.19 (1.51_2.64)	2.13 (1.75_2.73)	2.18 (1.71_2.83)
Glucose (mmol/L)	4.40 (4.20_4.90)	4.70 (4.30_5.20)	4.50 (4.20_4.90)
Lifestyle behaviors			
Smoking status			
Never smoked	44 (71)	211 (80)	240 (63)
Ever	18 (29)	52 (20)	140 (37)
Alcohol consumption			
Never	29 (47)	153 (58)	142 (37)
Ever	33 (53)	110 (42)	238 (63)
CVD measures (pulse wave velocity, PWV)			
At 12 months Median (IQR) (m/s)	6.80 (5.83_7.90)	7.30 (6.50_8.60)	7.00 (6.20_7.90)
HIV-related characteristics			
1st line ART		245 (93)	
2nd line ART		18 (7)	
Viral load (copies/mL)			
<50	14 (23)	195 (74)	
50–1000	8 (13)	21 (8)	
>1000	35 (56)	44 (17)	
Missing	5 (8)	3 (1)	
CD4^+^ cell count (cells/μL)			
<200	11 (18)	19 (7)	
200–349	12 (19)	49 (19)	
>349	34 (55)	189 (72)	

BMI, body mass index; BP, blood pressure; HDL, high-density lipoprotein; IQR, interquartile range; LDL, low-density lipoprotein; PWV, pulse wave velocity; SD, standard deviation.

Statistical measures: Mean (SD) reported for normally distributed continuous variables. Median (IQR) reported for skewed continuous variables. Percentages are shown for categorical variables.

**Fig. 2:**
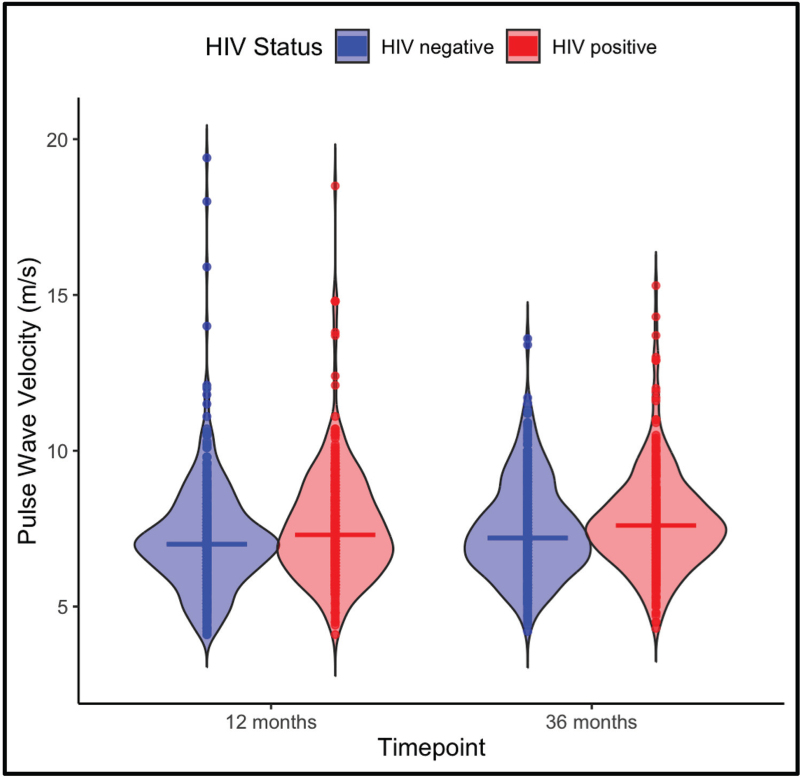
Pulse wave velocity (PWV) by HIV status at 12 and 36  months.

At 12  months, the median PWV was significantly higher among PWH (7.30  m/s) compared to HIV-negative people (7.00 m/s *P* = 0.001). From the 12  months to the 36  months timepoint, PWV increased significantly in both groups (*P* = 0.002) (Fig. 2). PWH on ART consistently exhibited the highest median PWV values at both time points. PWV increased from 7.3  m/s at 12  months to 7.6  m/s at 36  months in PWH on ART. The group of PWH who were ART-naïve at baseline showed the lowest median PWV (6.8  m/s) at 12  months, despite having initiated ART by this time point. However, this group demonstrated the largest increase in PWV, rising to 7.4  m/s at 36  months (*P* = 0.001). In contrast, the HIV-negative people group showed relatively stable PWV values, with an increase from 7.0  m/s at 12  months to 7.2  m/s at 36  months (Supplemantary Fig. 2, Supplemental Digital Content).

Violin plots display the distribution of PWV values for HIV-negative participants and people with HIV (HIV-positive). Horizontal colored lines represent group-specific medians at each time point.

Age and male sex had a positive significant association with PWV (in m/s) in all three groups (ART naïve at baseline, PWH on ART and HIV-negative people [0.13 (0.08_0.17), 0.09 (0.07_0.11), and 0.07 (0.05_0.08)] and [1.58 (0.36_2.80), 1.21 (0.76_1.67), and 0.63 (−0.21_1.04)] while BMI and educational attainment were inversely associated. Systolic and diastolic blood pressure were positively significantly associated with PWV in all groups, with triglycerides, total cholesterol, and smoking associated with PWV in PWH on ART (Table S2, Supplemental Digital Content).

In the mixed-effects model assessing change in arterial stiffness over time, follow-up time and HIV status showed a positive significant association with PWV. At 36  months, PWV was significantly higher compared to the 12-month timepoint, with an estimated increase of 0.31 m/s (*P* < 0.001). Age, male sex and SBP were positively significantly associated with PWV, with those being 49  years and older and being male showing highest increases (1.77  m/s and 0.49  m/s, respectively, *P* < 0.001). No significant associations were observed for education, smoking, or triglycerides, and no interaction between age and HIV status was found (Table 3).

**Table 3:** Multivariable mixed-effects model for predictors of pulse wave velocity (PWV) with interaction terms between Age groups and HIV status.

	Final adjusted model
	
	*β* (95% CI)
Time (visit)	
12 months	**Reference**
36 months	**0.31 (0.18_0.44)**
HIV status	
Negative	**Reference**
Positive	**0.65 (0.24_1.06)**
Age group	
18–29 years	**Reference**
30–49 years	**0.85 (0.55_1.15)**
>49 years	**1.77 (1.38_2.16)**
Sex	
Female	**Reference**
Male	**0.49 (0.28_0.69)**
Education	
<High school (HS)	**Reference**
HS	−0.36 (−0.78_0.03)
>HS	−0.36 (−0.78_0.06)
Smoking	
Never	Reference
Ever	0.06 (−0.16_0.28)
SBP (mmHg)	**0.01 (0.005_0.01)**
Triglyceride (mg/L)	0.10 (−0.03_0.23)
Interaction (age and HIV status)	
Age group (18–29 years * HIV negative)	**Reference**
30–49 years* HIV positive	−0.43(−1.04_0.17)
>49 years * HIV positive	−0.40 (−0.89_0.09)

Linear mixed-effects model with PWV (m/s) as the outcome. Values are *β* (95% CI). Reference groups: 12  months, HIV-negative, age 18–29  years, female, <high school education. Final adjusted model Includes time (visit), HIV status, age group, sex, education, systolic blood pressure, triglycerides, and interaction terms between age group and HIV status.

Furthermore, in a mixed model with ART status as the independent variable of interest, being on ART was significantly associated with PWV when compared to being HIV-negative, while ART-naive status at baseline showed no association with PWV. In the final adjusted model (Table 4), an interaction term between age and ART status indicated that among PWH aged >49  years, being ART-naive at baseline was significantly associated with higher PWV compared to HIV-negative individuals aged 18–29 years. Additionally, mixed models with interaction terms between HIV status and timepoint, as well as between ART status and timepoint, were run. However, neither of these interaction terms was significant (Table S3, Supplemental Digital Content and Table S4, Supplemental Digital Content).

**Table 4:** Multivariable mixed-effects model for predictors of pulse wave velocity (PWV) with interaction terms between age groups and ART status.

	Final adjusted model
	
	*β* (95% CI)
Time (visit)	
12 months	**Reference**
36 months	**0.31 (0.18_0.44)**
ART status	
HIV negative	**Reference**
ART naïve at baseline	−0.43 (−1.11_0.25)
On ART	**0.64 (0.10_1.19)**
Age group	
18–29 years	**Reference**
30–49 years	**0.86 (0.56_1.16)**
>49 years	**1.76 (1.37_2.15)**
Sex	
Female	**Reference**
Male	**0.51 (0.31_0.72)**
Education.	
<High school (HS)	**Reference**
HS	−0.24 (−0.51_0.02)
>HS	−0.33 (−0.75_0.09)
Smoking	
Never	Reference
Ever	0.06 (−0.16_0.29)
SBP (mmHg)	**0.01 (0.01_0.02)**
Triglyceride (mg/L)	0.06 (−0.05_0.16)
Interaction (ART status: age group)	
HIV negative* 18-29 years	**Reference**
ART naïve*30-49 years	0.54 (−0.04_0.16)
On ART*30-49 years	−0.35 (−0.96_0.26)
ART naïve*>49 years	**1.19 (0.11_2.26)**
On ART*>49 years	−0.01(−0.70_0.68)

Linear mixed-effects model with PWV (m/s) as the outcome. Values are *β* (95% CI). Reference groups: 12  months, HIV-negative, age 18–29  years, female, <high school education. Final adjusted model Includes time (visit), ART status, age, sex, education, systolic blood pressure, triglycerides, and interaction terms between ART status and age.

## Discussion

The results of our study provide important insights into the progression of arterial stiffness in PWH compared to HIV-negative people. PWV increased across all groups over time, with PWH showing higher PWV levels than HIV-negative people at both the 12- and 36-month time points in adjusted analyses. Among PWH, those who were ART-naïve at baseline had the lowest median PWV at 12  months, but this group experienced the largest increase by 36  months. Metabolic changes accompanying ART initiation may contribute to increases in arterial stiffness. The larger increase in PWV among ART-naïve participants compared to those already on ART highlights the importance of cardiovascular risk assessment and monitoring following HIV diagnosis.

The more stable PWV trajectory in the HIV-negative people group, with only a slight increase from 7.0  m/s to 7.2  m/s over 36  months, likely reflects the typical, gradual age-related arterial stiffening in this population. In contrast, the greater changes observed in the other groups may reflect the combined effects of HIV infection and ART on arterial stiffness. Our mixed-effects model, which is adjusted for confounders, emphasizes the significant role of both time and HIV status in predicting changes in PWV. The increase of 0.29  m/s over the 36  months among PWH suggests that HIV, combined with ART, has a lasting effect on vascular function. Age, male sex, and SBP also showed significant associations with higher PWV in our analysis, aligning with known risk factors for arterial stiffening.

Although the differences in PWV across groups were statistically significant, their clinical relevance warrants consideration. The estimated increase of 0.31 m/s over 36 months is below the commonly cited minimal clinically important difference of approximately 1.0–1.1 m/s [23,24]. Nevertheless, even smaller PWV increases may be meaningful in populations with a high burden of cardiovascular risk and inflammation, such as PWH. Importantly, these changes occurred over a relatively short follow-up period; continued increases at similar rates could accelerate the development of clinically relevant arterial stiffness and cardiovascular risk over time.

The large increase in PWV among ART-naïve participants, compared to those already on ART, underscores the importance of early cardiovascular risk monitoring in PWH. These findings align with previous research, such as a study conducted in Malawi, where PWH exhibited rapid increases in PWV, particularly during the first three months of ART initiation in participants with advanced HIV disease [25]. Moreover, studies investigating flow-mediated dilation and other cardiovascular outcomes have consistently shown elevated CVD risk in HIV-positive individuals [26,27]. These findings further strengthen the evidence linking HIV, ART, and increased arterial stiffness. Similar results have been reported, with higher PWV observed in ART-naïve PWH compared to HIV-negative people in a case-control study conducted in the Western Cape, South Africa [28]. Our longitudinal results expand on this by showing how PWV changes over time after ART initiation. This pattern is also supported by previous cross-sectional data from the Ndlovu cohort, which found that ART use was associated with increased carotid intima-media thickness (CIMT) in participants aged 30  years and older, with the association becoming stronger with older age [18]. The small sample size in the subgroup of ART-naïve individuals who initiated ART after enrolment is a limitation, and these results should therefore be interpreted with caution. Unsurprisingly, there were significantly more ART-naïve participants with CD4 counts <350 at baseline, and reconstitution of individuals with low CD4 counts has been associated with higher immune activation [29], which is linked to an accelerated risk of cardiovascular disease [30,31]. Although HIV and ART status can change over time, the number of seroconverters and ART initiators in our cohort was very small. In the subset analyzed, 26 participants (8%) seroconverted from HIV-negative to HIV-positive over the 36-month follow-up. All of these participants were ART-naïve at the first positive visit and were therefore considered ART-naïve at baseline. Participants classified as “on ART” had already been receiving treatment at study enrolment. Given the minimal number of time-varying changes, we treated HIV and ART status as fixed baseline variables in the analyses. While this approach simplifies the analysis, it is unlikely to substantially affect the study findings.

Interestingly, although our findings on PWV align with several studies, they contrast with others examining PWV and various CVD risk markers. A South African cross-sectional study by Phalane *et al.* found that vascular markers such as PWV, CIMT, and pulse pressure amplification (PPA) were comparable between PWH and HIV-negative people [16]. Similarly, Fourie *et al.* reported no significant difference in PWV between PWH and HIV-negative people, while Monteiro *et al.* found comparable results for PWV, although they did report significant associations with age, male sex, and SBP [32], consistent with our findings. In contrast, studies utilizing other CVD markers show variability in cardiovascular outcomes. For example, longitudinal research using the Framingham risk score (FRS) indicated that HIV-negative people had a higher FRS at both baseline and follow-up [33], differing from our results where PWH had higher PWV. While FRS and PWV measure different aspects of cardiovascular health, these findings highlight the complexity of assessing CVD risk across different populations and markers. Additionally, a cross-sectional study conducted in the Northwest province, South Africa found no significant associations between HIV status and CIMT, central systolic BP, and central pulse pressure across age groups [34]. This highlights the variability in cardiovascular outcomes depending on the risk markers used. In line with these findings, Vos and colleagues also demonstrated that neither HIV nor ART was associated with CIMT or carotid distensibility in an urban African population [35], further challenging the assumption of HIV as a direct contributor to vascular dysfunction in all settings. The increased PWV observed in PWH, however, may partly reflect the metabolic effects of older ART regimens, such as stavudine and zidovudine, which were linked to lipodystrophy, insulin resistance, and dyslipidemia. Notably, these effects may persist long after the cessation of such treatments [36]. The variability in outcomes, such as differing effects of ART on PWV, may also be attributed to differences in study populations. For instance, many participants from the Ndlovu Cohort Study on stable ART were previously exposed to older regimens, while populations in high-income countries or some other Sub-Saharan African studies often have shorter ART durations and less exposure to these regimens. The European AIDS Clinical Society 2023 guidelines highlight the shift towards safer integrase strand transfer inhibitor-based ART regimens, which are less likely to cause severe metabolic complications. However, concerns about weight gain, particularly among female and Black populations, persist due to drug effects and immunological recovery [37]. These evolving treatment regimens underscore the need to consider ART history when evaluating cardiovascular risk in PWH and tailoring management strategies.

### Limitations and strengths

One limitation of this study is the loss-to-follow-up of 25% from baseline to 12  months and 38% from baseline to 36  months in the larger study. This attrition may introduce potential bias, as those lost to follow-up could differ systematically from those who remained, potentially underrepresenting participants with higher cardiovascular risk or less consistent healthcare engagement. Future studies should consider strategies to improve follow-up rates, such as enhanced participant retention efforts, to strengthen the robustness of longitudinal findings. While data on specific ART drugs were available, they were spread across numerous variables with sparse counts, making modelling at the drug class or regimen level infeasible. For this reason, we analyzed ART status as a binary variable (ART-naïve vs. on ART). Although this approach simplified the analysis, it limited our ability to explore potential differences between ART classes in relation to PWV. Additionally, because this analysis used an existing subsample of the NCS cohort, no a priori power calculation was performed. While a post hoc calculation could be conducted, its inferential value would be limited.

A key strength of this study is nevertheless its large sample size (705 participants), which provides a robust foundation for identifying differences in PWV between PWH and HIV-negative people, in contrast to other studies with smaller cohorts. Additionally, the longitudinal design with two follow-up time points (12 and 36  months) allows for the evaluation of changes in arterial stiffness over time, offering a clearer understanding of the progression of cardiovascular risks, especially in relation to ART initiation and HIV infection. This is especially important when examining long-term impacts, as cross-sectional studies may not fully reveal the temporal changes in cardiovascular risk. However, we acknowledge the need for cautious interpretation, particularly given the inherent complexities and limitations of the data.

## Conclusion

Our study underscores the persistent cardiovascular burden faced by PWH, even after ART initiation. The observed increase in PWV, a marker of arterial stiffness, among PWH suggests that while ART improves survival and quality of life, it may not fully prevent vascular ageing. This highlights the need for ongoing cardiovascular monitoring and interventions aimed at reducing CVD risk in this population. For clinical practice, we advocate for heightened vigilance in identifying and addressing cardiovascular risk in PWH, with emphasis on early detection and management of vascular changes, particularly after ART initiation.

## Acknowledgements

We extend our heartfelt appreciation to the study participants, as well as to our esteemed colleagues from the Ndlovu Research Consortium and Ndlovu Care Group, whose invaluable contributions made this research possible. We also wish to acknowledge the National Research Foundation/NUFFIC (reference no.: MND200727546905), the Foundation Study Fund for South African Students, a private bursary donation from an alumna of the School of Physiology in memory of colleagues at Wits Physiology, and Sub-Saharan Africa Consortium for Advanced Biostatistics (SSACAB) for their financial support to PSS. Additionally, we are grateful to the National Heart, Lung, and Blood Institute of the National Institutes of Health under Award Number UG3HL156388 (Integrating HIV and hEART health in South Africa) for support to K.K.G. and W.D.F.V. The contents of this manuscript are solely the responsibility of the authors and do not necessarily reflect the views of the National Institutes of Health. Finally, we express our gratitude to the Dutch AIDS Foundation, Dioraphte Foundation, De Grote Onderneming, and the University Medical Centre Utrecht for funding the Ndlovu Cohort Study, whose support has been instrumental in advancing our research.

**Author's contributions:** P.S.S. conducted the literature search, data analysis and was primarily responsible for drafting the manuscript. K.K.G., K.S., and A.G.V.S. provided critical guidance on the study design, data interpretation, and revisions to the manuscript. All authors (P.S.S., K.S., C.N.U., R.E.B., W.D., R.A.C., D.E.G., W.D.F.V., H.T., A.G.V.S., and K.K.G.) contributed to the critical review of the manuscript, and the final approval of the submitted version. Each author provided important intellectual content and contributed significantly to the refinement of the study findings and conclusions.

**Funding:** National Research Foundation/NUFFIC (MND200727546905), the Foundation Study Fund for South African Students, and Sub-Saharan Africa Consortium for Advanced Biostatistics (SSACAB) Dutch AIDS Foundation, Dioraphte Foundation, De Grote Onderneming, and the University Medical Centre Utrecht.

### Conflicts of interest

The authors declare there are no conflicts of interest.

## Supplementary Material

Supplemental Digital Content
