# Supplementary material for: HIV and ART status at baseline are associated longitudinally with increased pulse wave velocity: findings from the Ndlovu Cohort Study
Source: AIDS. 2026 Feb 3;40(5):638–47. doi: 10.1097/QAD.0000000000004428 (PMC13034736; doi:10.1097/QAD.0000000000004428)
Supplement: Supplemental Digital Content [file aids-40-638-s001.docx]

Supplementary materials:

**Supplementary table 1. Baseline characteristics of the full Ndlovu Cohort Study (n=1927) and the analytic sample (n=705).**

|  | **Full cohort (HIV+)**  **(n=887)** | **Full cohort (HIV-)**  **(n=1040)** | **PWV cohort (HIV+)**  **(n=325)** | **PWV cohort (HIV-)**  **(N=380)** |
| --- | --- | --- | --- | --- |
| **Age (mean, SD))** | 41.5 (10.4) | 36.4 (14.2) | 41.1 (10.2) | 36.5 (13.3) |
| 18-29 yrs. | 109 (12.3) | 451 (43.4) | 41 (12.6) | 153 (40.3) |
| 30-49 yrs. | 574 (64.7) | 350 (33.7) | 212 (65.2) | 143 (37.6) |
| >49 yrs | 204 (23) | 239 (23) | 72 (22.2) | 84 (22.1) |
| **Sex** |  | | | |
| Female | 529 (59.6) | 527 (50.7) | 217 (66.8) | 142 (37.4) |
| Male | 358 (40.4) | 513 (49.3) | 108 (33.2) | 238 (62.6) |
| **Socioeconomic characteristics** |  | | | |
| **Education** |  | | | |
| Less than high school (<HS) | 239 (26.9) | 221 (21.2) | 78 (24) | 69 (18.2) |
| High school/matric (HS) | 589 (66.4) | 711 (68.4) | 230 (70.8) | 275 (72.4) |
| Higher education (>HS) | 59 (6.7) | 108 (10.4) | 17 (5.2) | 36 (9.5) |
| **Employment** |  | | | |
| Unemployed | 241 (27.2) | 159 (15.3) | 86 (26.5) | 61 (16.1) |
| Employed | 611 (68.9) | 696 (66.9) | 224 (68.9) | 258 (67.9) |
| Other (student, retired or volunteer) | 35 (3.9) | 185 (17.8) | 15 (4.6) | 61 (16.1) |
| **Monthly income per person**  **Median (IQR)** | 360 (1224) | 355 (1250) | 354.8 (1106.25) | 341.7 (1312.5) |
| <648 ZAR | 522 (58.9) | 621 (59.7) | 195 (63.3) | 224 (63.3) |
| 648-992 ZAR | 65 (7.3) | 79 (7.6) | 23 (7.5) | 26 (7.3) |
| >992 ZAR | 248 (28) | 289 (27.8) | 90 (29.2) | 104 (29.4) |
| **Health status** |  | | | |
| BMI (mean, SD) | 23.6 (5.87) | 24.6 (6.22) | 41.1 (10.2) | 36.5 (13.3) |
| Systolic BP (mm Hg) | 115 (20.5) | 120 (24.1) | 114.0 (19.1) | 122.0 (23.9) |
| Diastolic BP (mm Hg) | 73.6 (12.9) | 74.8 (14.2) | 73.6 (12.2) | 75.2 (14.7) |
| Pulse rate/min | 75.0 (13.0) | 71.0 (12.9) | )77.0 (13.0) | 72.0 (14.0) |
| Triglycerides (mg/L) | 1.2 (0.8) | 1.1 (0.7) | 1.0 (0.7) | 0.9 (0.6) |
| Total cholesterol (mmol/L) | 4.26 (1.0) | 4.19 (1.0) | 4.1 (1.2) | 4.1 (1.2) |
| HDL (mmol/L) | 1.43 (0.43) | 1.38 (0.34) | 1.44 (0.52) | 1.38 (0.46) |
| LDL (mmol/L) | 2.30 (0.84) | 2.32 (0.89) | 2.13 (1.01) | 2.18 (1.12) |
| Glucose (mmol/L) | 4.85 (1.22) | 5.02 (2.65) | 4.7 (0.85) | 4.5 (0.70) |
| **Lifestyle behaviors** |  | | | |
| **Smoking status** |  | | | |
| Never smoked | 559 (63) | 581 (55.9) | 255 (78.5) | 240 (63.2) |
| Ever | 327 (36.9) | 459 (44.1) | 70 (21.5) | 140 (36.8) |
| **Alcohol consumption** |  | | | |
| Never | 306 (34.5) | 263 (25.3) | 182 (56) | 142 (37.4) |
| Ever | 581 (65.5) | 777 (74.7) | 143 (44) | 238 (62.6) |
| **CVD measures (pulse wave velocity, PWV)** |  | | | |
| At 12 months  Median (IQR): m/s | 7.30 (2.1) | 7.00 (2.0) | 7.3 (2.2) | 7.0 (1.7) |
| **HIV-related characteristics** |  | | | |
| Seroconveters | 51 (5.7) |  | 26 (8.0) |  |
| ART naive | 118 (13.3) |  | 36 (11.1) |  |
| 1^st^ line ART | 649 (73.2) |  | 245 (75.4) |  |
| 2^nd^ line ART | 67 (7.6) |  | 18 (5.5) |  |

ART: Antiretroviral therapy. BMI: Body mass index. BP: Blood pressure. HDL: High-density lipoprotein. LDL: Low-density lipoprotein. PWV: Pulse wave velocity. IQR: Interquartile range. SD: Standard deviation. ZAR: South African Rand (approximately 1 USD ≈ 18.4 ZAR at the time of study).

Statistical measures: Mean (SD) reported for normally distributed continuous variables. Median (IQR) reported for skewed continuous variables. Percentages are shown for categorical variables. Group comparisons were conducted using independent t-tests for normally distributed variables, Mann-Whitney U tests for non-normally distributed variables, and chi-square tests for categorical variables.

**Supplementary table 2: Bivariate analysis for 325 PLWH and 380 HIV-negative people with complete PWV data at 12 and 36 months (outcome=PWV)**

|  | PLWH (n=325) | | HIV-negative(n=380) |
| --- | --- | --- | --- |
|  | ART naïve at baseline (n=62) | On ART  (n=263) |  |
|  | β (95%CI) | β(95%CI) | β(95%CI) |
| AGE, yrs. | **0.13 (0.08_0.17)** | **0.09 (0.07_0.11)** | **0.07 (0.05_0.08)** |
| GENDER | | | |
| Female | Reference | | |
| Male | **1.58 (0.36_2.80)** | **1.21 (0.76_1.67)** | **0.63 (0.21_1.04)** |
| EDUCATION | | | |
| <High school | Reference | | |
| High school/matric (HS) | **-1.74 (-3.00_ -0.47)** | **-0.78 (-1.31_ -0.25)** | **-1.25 (-1.77_** **-0.74)** |
| Higher education (>HS) | -1.67 (-3.72_ 0.36) | **-1.50 (-2.68_ -0.31)** | **-1.16 (1.95_** **-0.38)** |
| EMPLOYMENT | | | |
| Unemployed | Reference | | |
| Employed | 0.11 (-1.31_ 1.54) | -0.01 (-0.51_ 0.50) | -0.46 (-1.00_ 0.08) |
| Other (student, retired, or volunteer) | 0.35 (0.35_ 0.35) | -0.15 (-1.34_ 1.03) | **-1.34 (-2.03_** **-0.64)** |
| MONTHLY INCOME PER PERSON | -0.0004 (-0.004_0.003 | 0.002 (-0.001_0.0001) | 0.004 (-0.001_0.01) |
| <648 ZAR | Reference | | |
| 648-992 ZAR | 2.14 (-2.49_6.77) | -0.07 (-0.93_0.77) | **-0.05 (-0.87_-0.87)** |
| >992 ZAR | 0.05 (-1.32_1.43) | 0.05 (-0.47_0.58) | -0.14 (-0.61_0.32) |
| Health status | | | |
| BMI ( kg/m^2^) | -0.02 (-0.11_0.07) | **-0.06 (-0.10_-0.02)** | 0.03 (-0.004_0.07) |
| Normal weight (18.5-24.9) | Reference | | |
| Underweight (<18.5) | 1.01 (-0.55_2.57) | 0.25 (-0.41­_0.92) | -0.18 (-0.78_0.41) |
| Overweight (25-29.9) | -0.30 (-1.87_1.25) | -0.57 (-1.16_0.02) | -0.26 (-0.79_0.25) |
| Obese (>30) | 0.83 (-0.93_2.60) | -0.95 (-1.60_-0.30) | 0.47 (-0.12_1.08) |
| Systolic BP (mmHg) | **0.03 (0.01_0.06)** | **0.03 (0.02_0.04)** | **0.02 (0.01_0.03)** |
| Diastolic BP (mmHg) | **0.08 (0.03_0.12)** | **0.04 (0.02_0.06)** | **0.03 (0.02_0.04)** |
| Pulse rate per min (beats/min) | -0.01 (-0.05_0.03) | -0.001 (-0.02_0.02) | 0.004 (-0.01_0.02) |
| Triglycerides (mg/L) | 0.73 (-0.65_2.12) | **0.28 (0.01_0.55)** | **0.39 (0.11_0.67)** |
| Total cholesterol (mmol/L) | 0.40 (-0.20_1.01) | **0.32 (0.08_0.56)** | **0.23 (0.03_0.44)** |
| HDL ( mmol/L) | 1.02 (-0.51_2.55) | 0.46 (-0.09_1.02) | 0.38 (-0.18_0.96) |
| LDL ( mmol/L) | 0.20 (-0.44_0.86) | 0.11 (-0.17_0.39) | 0.12 (-0.10_0.34) |
| Glucose ( mmol/L) | -0.25 (-1.24_0.73) | **0.18 (0.002_0.37)** | 0.04 (-0.05_0.15) |
| SMOKING STATUS | | | |
| Never smoked | Reference | | |
| Ever | 0.71 (-0.53_1.96) | **0.66 (0.10_1.23)** | 0.21 (-0.19_0.63) |
| ALCOHOL CONSUMPTION | | | |
| Never | Reference | | |
| Ever | -0.08 (-1.23_1.06) | 0.07 (-0.38_0.53) | **-0.53 (-0.94_-0.12)** |
| Viral load copies/mL | | | |
| <50 | Reference | | |
| 50-1000 | **2.70 (0.34_5.06)** | 0.87 (-0.06_1.80) |  |
| >1000 | -0.42 (-1.67_0.81) | -0.07 (-0.92_0.77) |  |
| CD4 count ( cells/μL) | | | |
| <200 | Reference | | |
| 200-349 | **-1.89 (-3.64_-0.14)** | -0.67 (-1.66_0.32) |  |
| >349 | -1.22 (-2.67_0.23) | **-1.05 (-1.93_-0.16)** |  |

β coefficients represent the change in pulse wave velocity (PWV, m/s) per unit increase in the predictor variable (continuous variables) or relative to the reference category (categorical variables), with 95% confidence intervals (CI) shown in parentheses. Analyses were conducted separately for ART-naïve PLWH, PLWH on ART, and HIV-negative , using complete PWV data at 12 months.


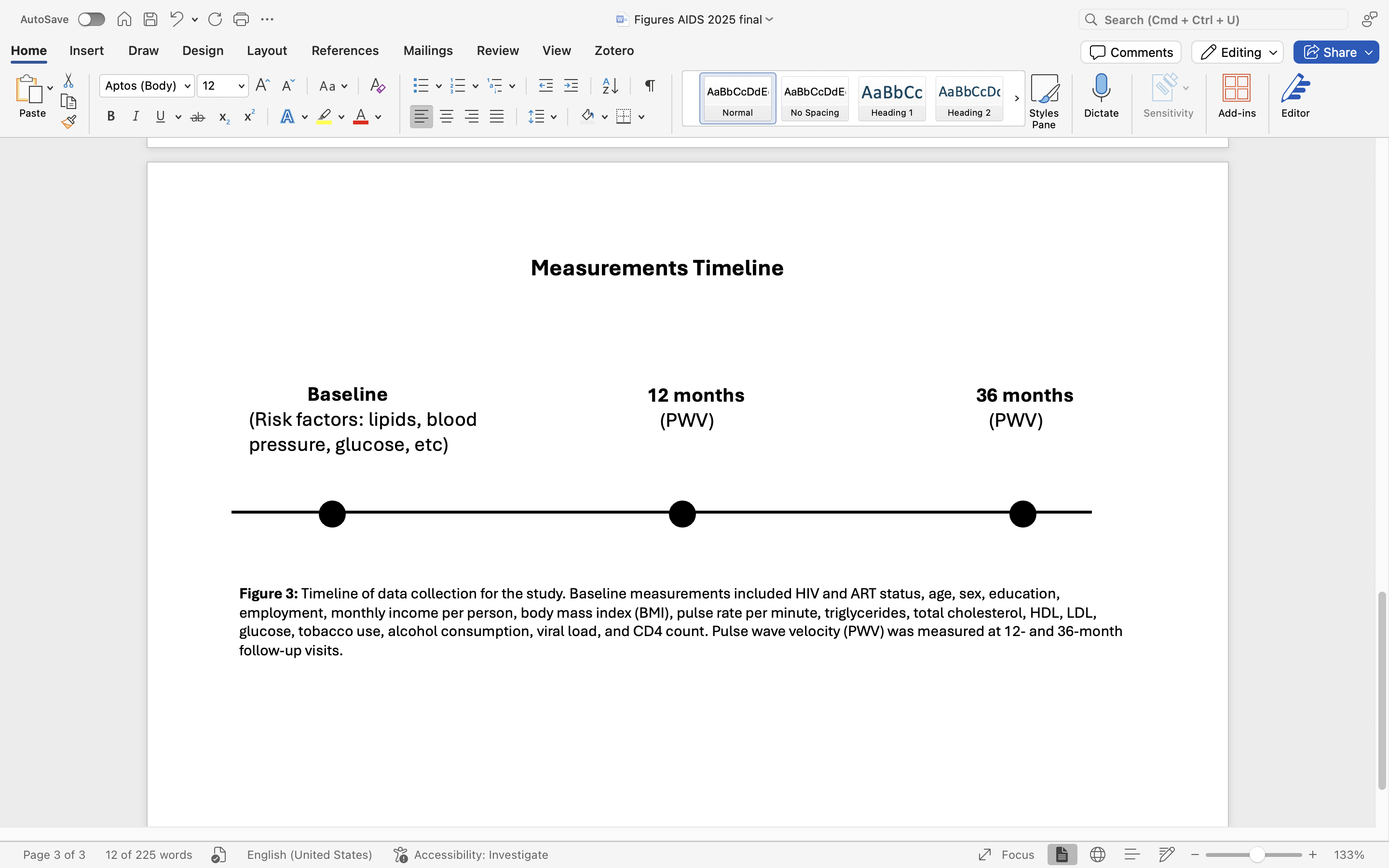


**Supplemantary Figure 1:** Timeline of data collection for the study. Baseline measurements included HIV and ART status, age, sex, education, employment, monthly income per person, body mass index (BMI), pulse rate per minute, triglycerides, total cholesterol, HDL, LDL, glucose, tobacco use, alcohol consumption, viral load, and CD4 count. Pulse wave velocity (PWV) was measured at 12- and 36-month follow-up visits.

**
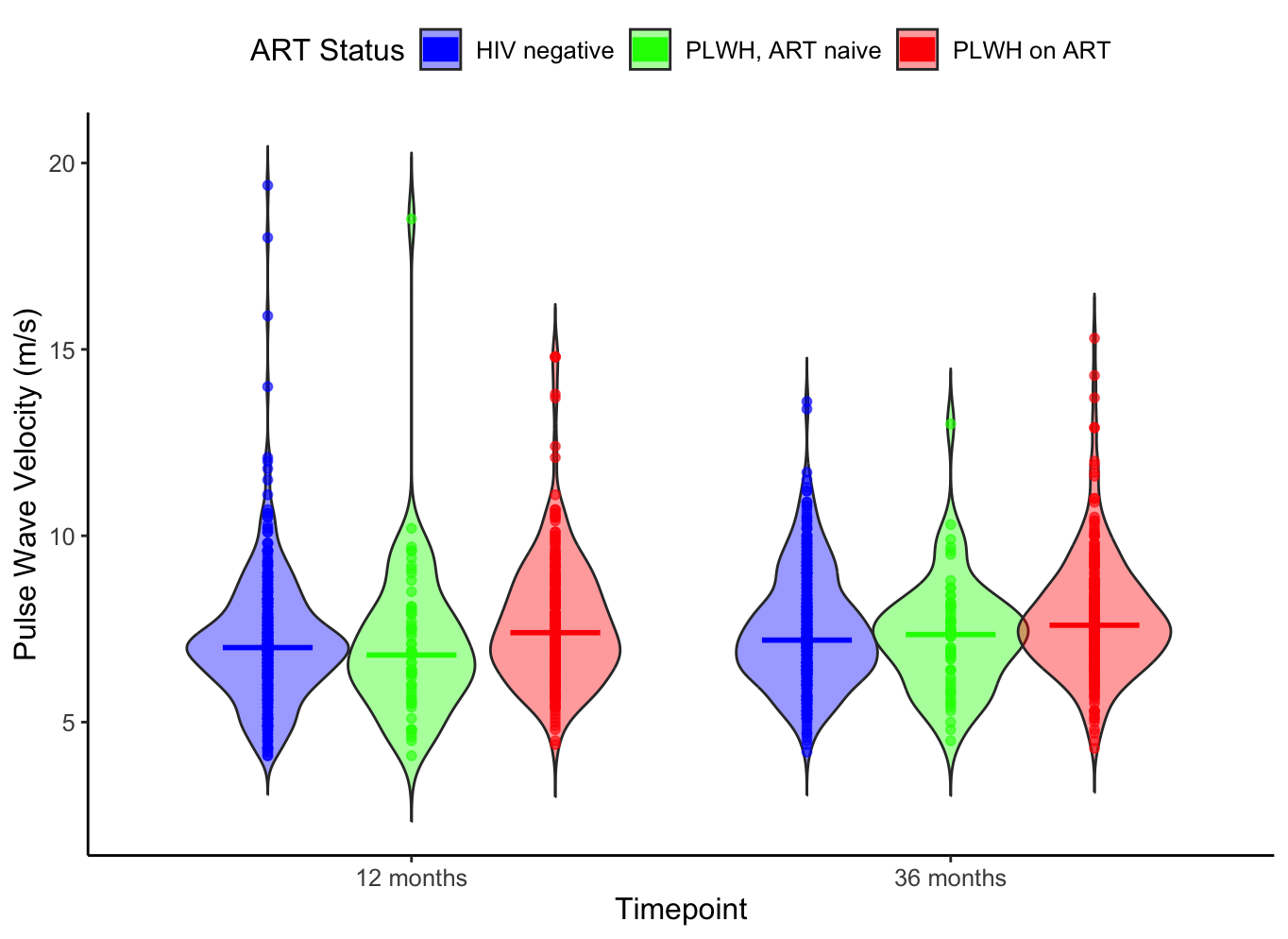
**

**Supplemantary Figure 2:** Pulse wave velocity by ART status at 12 and 36 months. Violin plots show PWV distributions for HIV-negative participants, PLWH who were ART-naïve at baseline, and PLWH on ART. Horizontal coloured lines indicate the group-specific medians within each time point.

**Supplementary Table 3: Multivariable mixed-effects model for** **predictors of Pulse Wave Velocity (PWV) with interaction terms between HIV status and timepoints (visits)**

|  | **Model** |
| --- | --- |
|  | β(95% CI) |
| **Time (visit)** |  |
| 12 months | **Reference** |
| 36 months | **0.36 (0.18_0.54)** |
| **HIV status** |  |
| Negative | **Reference** |
| Positive | **0.41 (0.15_0.66)** |
| **Age group** |  |
| 18-29 yrs | **Reference** |
| 30-49 yrs | **0.82 (0.57_1.07)** |
| >49 yrs | **1.94 (1.61_2.27)** |
| **Sex** |  |
| Female | **Reference** |
| Male | **0.47 (0.25_0.70)** |
| **Education** |  |
| <High school (HS) | **Reference** |
| HS | -0.25 (-0.51_0.02) |
| >HS | -0.37 (-0.79_0.06) |
| **Smoking** |  |
| Never | **Reference** |
| Ever | 0.07 (-0.15_0.29) |
| **SBP (mmHg)** | **0.01 (0.001_0.01)** |
| **Triglyceride (**mg/L) | 0.10 (-0.04_0.23) |
| **Interaction (HIV status and Timepoints)** |  |
| Negative: 12 months | **Reference** |
| Positive: 36 months | -0.11 (-0.38_0.15) |

Reference categories for the mixed-effects model were: 12 months for time, HIV-negative for HIV status, age 18-29 years, female sex, <high school education, and never smoking. Systolic blood pressure (SBP) and triglycerides were modelled as continuous variables. The interaction term between HIV status and time was parameterised with HIV-negative at 12 months as the reference group.

**Supplementary Table 4: Multivariable mixed-effects model for predictors of PWV with interaction terms between ART status and timepoints (visits)**

|  | **Model** |
| --- | --- |
|  | β(95% CI) |
| **Time (visit)** |  |
| 12 months | **Reference** |
| 36 months | **0.36 (0.18_0.54)** |
| **HIV status** |  |
| Negative | **Reference** |
| ART naive | 0.12 (-0.31_0.55) |
| On ART | **0.49 (0.23_0.76)** |
| **Age group** |  |
| 18-29 yrs | **Reference** |
| 30-49 yrs | **0.81 (0.56_1.06)** |
| >49 yrs | **1.89 (1.55_2.23)** |
| **Sex** |  |
| Female | **Reference** |
| Male | **0.49 (0.26_0.71)** |
| **Education** |  |
| <High school (HS) | **Reference** |
| HS | -0.26 (-0.53_0.01) |
| >HS | -0.36 (-0.78_0.07) |
| **Smoking** |  |
| Never | **Reference** |
| Ever | 0.09 (-0.13_0.31) |
| **SBP (mmHg)** | **0.01 (0.01_0.02)** |
| **Triglyceride (**mg/L) | 0.09 (-0.04_0.22) |
| **Interaction (ART status and Timepoints)** |  |
| Negative: 12 months | **Reference** |
| ART naïve: 36 months | -0.04 (-0.52_0.43) |
| On ART: 36 months | -0.13 (-0.41_0.15) |

Linear mixed-effects model with PWV (m/s) as outcome. Values are β (95% CI). Reference groups: 12 months, HIV-negative, age 18–29 years, female, <high school education, never smoking. Model adjusted for time, HIV/ART status, age, sex, education, SBP, triglycerides, and ART × time/visit interaction.
